# Behaviour Change Considerations to Promote Physical Activity Participation among Individuals with Quiescent Inflammatory Bowel Disease: Barriers and Facilitators

**DOI:** 10.1177/27536351251382074

**Published:** 2025-10-20

**Authors:** Banke Oketola, Sandra Webber, Harminder Singh, Maia Kredentser, Kristin Reynolds, Gayle Restall

**Affiliations:** 1 Applied Health Sciences Program, Faculty of Graduate Studies, University of Manitoba, Winnipeg, MB, Canada; 2 Department of Internal Medicine, Max Rady College of Medicine, Rady Faculty of Health Sciences, University of Manitoba, Winnipeg, MB, Canada; 3 Department of Physical Therapy, College of Rehabilitation Sciences, University of Manitoba, Winnipeg, MB, Canada; 4 Department of Psychology and Department of Psychiatry, University of Manitoba, Winnipeg, MB, Canada; 5 Department of Occupational Therapy, College of Rehabilitation Sciences, University of Manitoba, Winnipeg, MB, Canada

**Keywords:** inflammatory bowel diseases, quiescent IBD, physical activity participation, behaviour change, COM-B model

## Abstract

**Background:**

Most individuals with inflammatory bowel diseases (IBD) do not engage in optimum levels of physical activity (PA). This study aimed to identify important factors to consider when promoting PA participation among individuals with quiescent or mildly active IBD.

**Methods:**

In this qualitative description study, we purposively enrolled 15 Manitobans with quiescent IBD. Data was collected via semi-structured interviews conducted on Zoom. Using the Capability, Opportunity, Motivation, and Behaviour (COM-B) model, we elicited factors that influence PA behaviour. We performed thematic analysis of transcribed interviews using NVivo.

**Results:**

Participants’ (N = 15) ages ranged between 20 and 37 years, majority were female (n = 8), and most had a diagnosis of ulcerative colitis (UC) (n = 10). None was a current smoker. Thematic analysis identified persistent symptoms, PA engagement prior to IBD diagnosis, PA routine, coping strategies and determination as themes that influenced ‘capability’ for PA participation. Social support, type of employment, bathroom access, and finances influenced ‘opportunity’. Awareness of the benefits of PA on IBD, relevant knowledge, social support, and personal goals promoted ‘motivation’ for PA participation. Sex differences were noted in participants’ perspectives related to safety concerns and the social context of workout spaces.

**Conclusion:**

Using the COM-B model, we elicited barriers (including IBD-related persistent symptoms, lack of knowledge of PA) and facilitators (social support, coping strategies, ability to track PA progress) that influence PA behaviour among adults with quiescent IBD. These factors are important considerations when promoting PA participation among individuals with IBD.

## Introduction

Inflammatory bowel disease (IBD) refers to remitting and relapsing inflammatory conditions of the gastrointestinal (GI) tract.^1^ IBD usually presents as either Crohn’s disease (CD) where the inflammation can be anywhere from the mouth to the anus, or as ulcerative colitis (UC) where the inflammation is in the large bowel only.^2^ IBD is a lifelong condition characterized by periods of active inflammation requiring medical and/or surgical therapies to relieve acute symptoms and achieve mucosal healing; and periods of inactive disease (i.e. quiescent disease) requiring regular maintenance medical therapies.^1,3^ Even though the disease burden is worse during periods of active disease, individuals with quiescent IBD, that is, IBD in remission, frequently report a number of physical and psychological symptoms.^3^ Physical symptoms often include fatigue, abdominal pain, joint pain; and psychological symptoms can include depression, worry, stress, and anxiety.^4-8^ Extraintestinal manifestations of IBD, which include arthritis, erythema nodosum, iritis and uveitis, low bone mineral density, and loss of muscle mass, can affect up to 25% of patients.^9,10^ Additionally, IBD drug therapies can cause side effects leading to some persistent symptoms.^11^ For example, prolonged prednisone use can lead to a higher chance of developing osteoporosis, with consequent joint pain, osteoporotic fractures, and muscle weakness.^10,12^ Due to ongoing symptoms, individuals with quiescent IBD continue to seek adjunct therapies in addition to medical therapies to manage their disease and improve their quality of life (QoL).^1,13^

Physical activity (PA) and exercise have been used as a form of adjunctive therapy for a variety of chronic diseases.^14,15^ The World Health Organization (WHO) defines PA as ‘any bodily movement produced by skeletal muscles that requires energy expenditure and can be performed at a variety of intensities, as part of work, domestic chores, transportation or during leisure time, or when participating in exercise or sports activities’^16^ (p. 15). Exercise on the other hand, is defined by WHO as a ‘subcategory of physical activity that is planned, structured, repetitive, and purposeful in the sense that the improvement or maintenance of one or more components of physical fitness is the objective’^16^ (p. VI). In this study, we use PA generally to encompass all activities that can be engaged in to improve fitness, flexibility, and cardiorespiratory endurance, such as structured exercise, walking, yoga, swimming, etc. The WHO recommends that adults 18 to 65 years perform 150 to 300 minutes of moderate to vigorous intensity PA to gain substantial health benefits.

In IBD, several studies have highlighted that PA could be a potential adjunct therapy to medical therapy to help reduce ongoing symptoms in quiescent disease state and improve quality of life (QoL).^3,8,14,17,18^ PA has been recommended because it may improve immunological response, counteract some IBD-specific complications, including reversing reductions in muscle mass and bone mineral density, as well as reduce anxiety and fatigue.^3,19,20^ However, despite the potential benefits of PA, a large proportion of individuals with IBD do not regularly participate in PA or meet PA guidelines set out for individuals with chronic conditions.^19^

It may be challenging to encourage people with quiescent IBD to engage in optimal levels of PA because it is a complex behaviour change that involves significant commitment, complicated by ongoing symptoms, and lack of knowledge of the benefits of specific types of PA^21-23^ Additionally, there are no PA recommendations or guidelines for IBD.^24,25^ Furthermore, a few cross-sectional studies investigating PA engagement among adults with IBD report higher PA engagement among males compared to females.^26-28^ Even though there are a number of papers investigating the effects of different forms of PA in the IBD population,^8,14,29^ and those highlighting the value of PA for members of this group,^17,24,30^ there is a scarcity of research exploring the barriers to PA participation and potential facilitators of participation, from the patient perspective. Eliciting the perspectives of individuals with IBD, their experiences with PA and factors that influence participation, such as socioeconomic parameters and correlates of PA, including employment, age, sex, gender, and smoking status, may provide relevant information necessary to understand PA behaviour, and factors that influence behaviour change among individuals with quiescent IBD. This is important to design or plan functional and effective PA programs that increase PA engagement among individuals with quiescent IBD.^22^

The purpose of this study was to explore patient experiences with PA, so as to elicit the factors that influence PA participation among adults aged 20 to 45 years with quiescent IBD who continue to experience persistent symptoms. Our primary objective was to explore the experiences of PA participation among a sample of adults living with quiescent IBD to elicit the factors that influence their PA participation. The secondary objective was to explore the sex differences in perspectives and preferences for PA among individuals with quiescent IBD.

## Materials and Methods

### Study Design

This was a qualitative description study.^31^ Even though we utilized a qualitative description approach, we employed some degree of interpretation at the level of data analysis and data interpretation.^32^ To be transparent with our analytical processes, we define our theoretical approach, lens and assumption.

### Theoretical Framework

The Capability, Opportunity, Motivation and Behaviour (COM-B) model suggests that a voluntary behaviour (B) is dependent on the presence of capability (C), opportunity (O), and motivation (M); and the occurrence of the behaviour (B) in turn influences the 3 conditions.^33^ Capability can be defined as the extent of someone’s ability or the power to do something, and is both physical and psychological.^34^ Opportunity, which can be both physical and social, refers to a favourable set of circumstances.^34,35^ Motivation, on the other hand, is a complex psychological process that focuses on achieving pre-set goal(s), including both the initiation and maintenance of efforts to achieve the goal(s).^36^ As explained by Michie and colleagues,^35^ for an individual to perform a target behaviour (B) at any given time, they must possess the physical and psychological capability (C) to do so, be armed with social and physical opportunities (O) and possess automatic and reflective motivation (M) to follow through; that is, they must want to perform that behaviour more than any other behaviour in that moment. In this study, performing at least 150 minutes of moderate to vigorous PA was the target behaviour or desired behaviour.

### Philosophical Assumptions

We utilized the constructivist paradigm in this study. This paradigm posits that realities are the products of interactions of the human with the real world, thus knowledge generation must be approached from multiple perspectives.^37^ To establish the validity of our study results, we used the participant lens, which focuses on checking that the participants’ realities are accurately represented in the final account (e.g. member checking).^37,38^

This study was approved by the University of Manitoba Health Research Ethics Board. All participants provided signed informed consent. We performed our reporting according to the Consolidated Criteria for reporting qualitative research (COREQ).^39^

### Study Population

Participants for this study were Manitobans, 20 to 45 years, with quiescent IBD. The experiences adults have can be quite different from those of adolescents and older adults. And these differences may influence the way they experience IBD and incorporate PA into their lives. The age limit was applied to reduce participant variance; thus, we focused on the peak age group of IBD onset. Only those who have not had active disease in the preceding 6 months but had at least one of the persistent symptoms of interest – abdominal pain, joint pain, fatigue, stress, anxiety, and depression, despite remission were eligible. Due to COVID social distancing protocols that were still in place, all interviews were completed via videoconferencing. Therefore, all eligible participants were required to have access to supports for videoconferencing.

### Participant Recruitment

We recruited participants via maximum variation purposive sampling based on age, gender, and IBD diagnosis. This sampling technique is aimed at intentionally selecting individuals with similar characteristics but different, individually nuanced experiences that are relevant to fulfil the study objectives.^40^ Participants ages 20 to 45 years old with CD or UC in remission for at least 6 months, were recruited from a large teaching hospital, gastroenterology clinics in the community, and through the Crohn’s and Colitis Canada-Manitoba Chapter. We acknowledge that it may be impossible to achieve saturation wholly;^41^ however, we wanted to approach the decision on when to stop data collection in a systematic manner. To ensure we obtained robust data to achieve study objectives within research resources, we followed the recommendations of Francis and colleagues^42^ for establishing study-specific data saturation. The research team contacted N = 22 potential participants via telephone. Seven were not enrolled due to scheduling conflicts or not meeting eligibility criteria. We purposively enrolled 5 male and 5 female participants as our initial analysis sample. We performed data analysis to identify new information and shared meanings. Upon confirming that we continued to obtain new information in the data, we continued participant enrolment in increments of 2, while concurrently performing data analysis with each set of additional data. We found new data was no longer recurring at participant number 12. We enrolled 2 more participants (stopping criterion) to confirm this. We enrolled 1 more participant with CD to ensure our study sample was more balanced in relation to the distribution of participants with UC and CD.

### Data Collection

We collected demographic and socioeconomic information from participants. Qualitative data was collected using semi-structured interviews completed via videoconference (Zoom, version 6.3.11), with only 1 participant and 1 researcher (BO) in each session. Each participant interview was conducted following the interview guide. Interview questions were organized around the COM-B model. The interview questions were developed and refined to elicit capability, opportunity, and motivation factors that influence PA participation by participants. Prior to the first interview, the interview guide was reviewed by 2 patient-partners to ensure comprehensiveness and relevance of the questions. The refined interview guide was pilot-tested with an individual living with a chronic condition prior to commencement of data collection. At each interview, we asked about perspectives on PA participation, their personal experiences with PA engagement, and factors that limited or enhanced their PA participation. The interviews were recorded using Zoom’s record feature and transcribed verbatim by a transcription service. To ensure data accuracy, transcripts were verified against original video files by a member of the research team (BO), after which the video files were deleted.

### Data Analysis

Demographic information is presented as counts and percentages. We used NVivo Qualitative Data Analysis software to organized and analyse the transcripts. Using coding reliability thematic analysis with a focus on description,^43^ we developed the initial codebook using the research objectives. One member of the research team (B.O.) read all the transcripts, analysed the first transcript and refined the coding framework. Another member of the research team (G.R.) reviewed the coded data and coding framework to ensure consistency and accuracy. After review and refinement of the coding framework, analysis of all subsequent transcripts was completed by B.O. and further reviewed by G.R. Discrepancies were resolved through discussion. Our analysis was driven first by the research objectives to generate broad themes. Next, we performed fine-coding to develop significant patterns of shared meanings relevant to our understanding of the study findings. Additional sub-themes were formulated from themes within the data and to provide more concise descriptions.

All themes were then organized under the 3 key headings of the theoretical framework – capability, opportunity, and motivation. This organization facilitated the use of the COM-B model to improve our understanding of the factors that influence PA participation among individuals with quiescent IBD. To ensure trustworthiness and rigour, participants were provided with a summary of study results and asked to verify that their perspectives had been represented. Participants approved the summary, and no further modifications were made. The final results were shared with a patient advisory committee. They confirmed that they could relate with the study results as adults living with IBD themselves (which enhanced the validity of our findings), they recommended using ‘fitness trackers’ instead of using the word ‘gadgets’, and they recommended using visualizations for the results. Their feedback enhanced our understanding and interpretation of study findings.

## Results

Between March and July 2023, we interviewed 15 adults with persistent symptoms in quiescent IBD (Table 1). Fatigue was the most frequently experienced persistent symptom, with 13 participants (87%) experiencing it at least sometimes (including ‘often’ and ‘always’); followed by stress (80%), anxiety (73%), depression (53%), abdominal pain (53%) and lastly, joint pain (33%) (Table 2). Interviews lasted between 35 and 60 minutes.

**Table 1.** Demographic Characteristics.

Variable	N (%)
Age	
20-24	4 (27)
25-29	8 (53)
30-34	2 (13)
35-40	1 (7)
IBD Diagnosis	
CD	5 (33)
UC	10 (67)
Sex	
Female	8 (53)
Male	7 (47)
Gender	
Female	8 (53)
Male	6 (40)
I prefer not to answer	1 (7)
Marital Status	
Single – never married	7 (47)
Common-law	4 (27)
Married	3 (20)
My relationship status is not listed here	1 (7)
Highest level of education	
Post-graduate degree	1 (7)
College/university degree	10 (67)
High school degree	3 (20)
Less than high school	1 (7)
Household income	
Less than $CAD 25,000 per annum	4 (27)
$CAD 25,001 to $CAD 50,000 per annum	4 (27)
$CAD 50,001 to $CAD 75,000 per annum	2 (13)
$CAD 75,001 to $CAD 100,000 per annum	3 (20)
More than $CAD 100,000 per annum	2 (13)
Smoking status	
Never smoker	12 (80)
Previous smoker	3 (20)
Current smoker	0 (0)
IBD Duration	
1-2 years	5 (33)
3-5 years	4 (27)
>5 years	6 (40)

Abbreviations: CD, Crohn’s disease; IBD, Inflammatory Bowel Disease; N, number of participants; UC, Ulcerative Colitis.

**Table 2.** Frequency and Impact of Persistent Symptoms.

Frequency	Fatigue	Abdominal pain	Joint Pain	Stress	Anxiety	Depression
Never	1	4	5	1	1	5
Seldom	1	3	5	2	3	2
Sometimes	7	5	4	0	1	5
Often	5	3	1	10	9	3
Always	1	0	0	2	1	0
Impact	Fatigue	Abdominal pain	Joint pain	Stress	Anxiety	Depression
Not at all	3	6	9	3	4	7
A little	1	4	4	3	2	2
Medium	5	3	1	2	2	2
A lot	6	2	1	4	4	2
Extremely	0	0	0	3	3	2

### Experiences with PA Participation

We used the COM-B model to organize study findings under the headings Capability, Opportunity and Motivation. Based on this organization, we detailed participants’ experiences with performing PA. From their experiences, we explored the factors that influence optimal PA participation (the desired behaviour) among individuals with quiescent IBD (target population). Of note, some themes were identified that influenced more than one of the COM components. We describe each of the themes below with supporting quotes from participants. Quotes are labelled by the participant’s sex, IBD diagnosis and study number.

**Figure 1.**
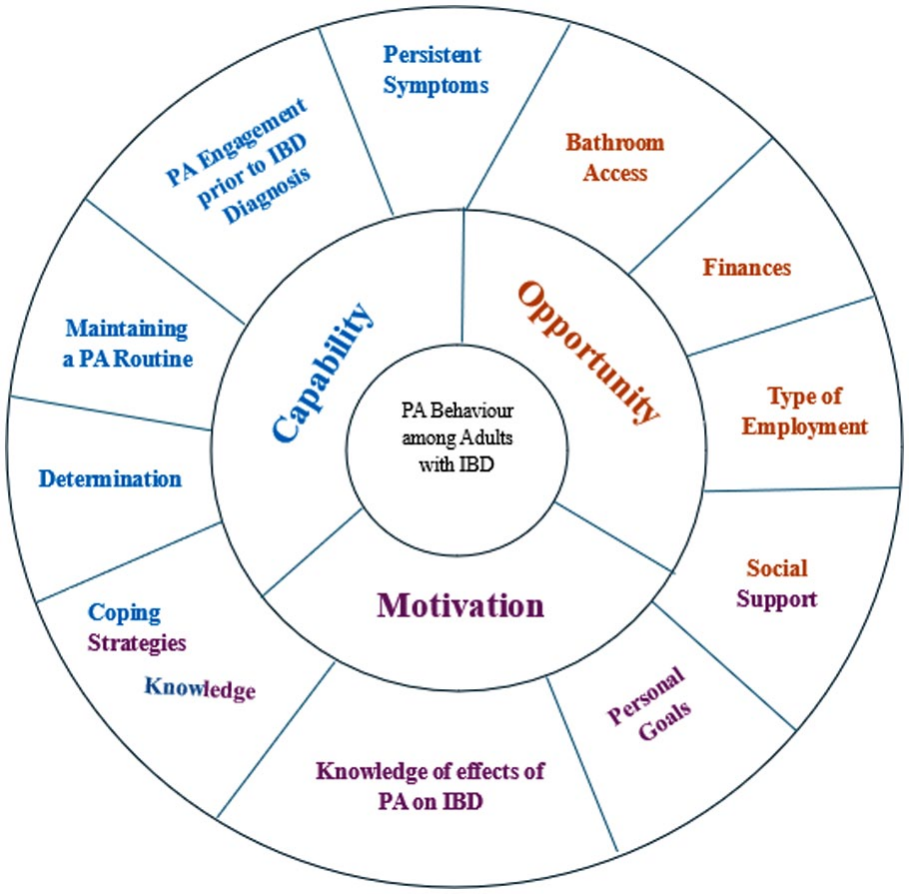
COM factors to promote physical activity behaviour change among adults with quiescent IBD.

Figure 1 summarizes the ‘capability’, ‘opportunity’, and ‘motivation’ factors that influenced PA participation among adults with IBD as identified in this study. In this figure, we also highlight that some factors impacted more than one of the COM-B components, for example, social support impacted both ‘opportunity’ and ‘motivation’ for PA participation and ‘knowledge influenced both ‘motivation’ and ‘capability’.

*Capability*: We identified 5 themes that influenced ‘capability’, that is, the extent of someone’s physical and psychological ability or power to engage in PA. They are **persistent symptoms, PA engagement prior to IBD diagnosis, maintaining a PA routine, coping strategies and determination**:

*Persistent symptoms*: When discussing their challenges with engaging in PA, the majority of participants highlighted ongoing symptoms despite clinical remission. For example, fatigue, pain (joint pain, abdominal/core pain), and anxiety. All participants reported their specific persistent symptom has often reduced their ability to be as physically active as they would have wanted to be:

*Whether you are doing push ups or running, even walking, you always use your core muscles. And I guess if you have Crohn’s or colitis, you already have more pain there. Male, CD, #001.*

*I guess my fatigue is definitely something that I find is very challenging to overcome. Fatigue is a big one. . . It’s something that you always have to fight through. Female, CD, #012.*

An important sub-theme we identified related to persistent symptoms was participants’ lack of knowledge on the effect of PA on these symptoms. Some participants reported experiencing anxiety when considering PA participation, due to not knowing whether it could aggravate their ongoing symptoms. A few participants reported that due to not knowing, they chose to abstain from performing PA:

*And it's a lot, I mean I feel like it's just the same with like living with IBD in general, like it's a lot of trial and error in things like, whether it's food, or whether it's exercise or anything like that. And like sometimes trial and error gets to be too much. So, sometimes you just have to. . .take a step back. Female, CD, #007.*

*PA engagement prior to IBD diagnosis*: When discussing their PA engagement, some participants reported that being physically active prior to getting their IBD diagnosis facilitated their willingness and capability to engage with PA despite ongoing symptoms:

*Well, before I was like sick, I was pretty physical. So, I managed to have like a good starting point for when I was recovering. . .I didn't find it challenging to get back into exercising. I think. Yeah, I mean it wasn't too hard for me. Man, CD, #001*

From this discussion, we found that those who were less physically active prior to their IBD diagnosis tended to be more cautious with PA engagement due to fear of worsening their symptoms, which consequently reduced their ‘capability’ for PA participation:

*No, I think at the start of everything you need a certain person, like a professional’s advice. I don’t want to do online exercises and the next day I’ll be like, oh, my back hurts. So, I just want to get professional advice at the start and then I’ll make my to-do list. Female, UC, #009*

*Maintaining a PA routine*: Some participants with an existing PA routine reported finding it less challenging to stay physically active:

*Yeah, I think to get back into it, even just walking, there wasn't a lot of anxiety. Female, CD, #005.*

Interestingly, some other participants reported being unable to establish a routine often due to the unpredictability of IBD:

*I think the problem currently with my routine is like the way I'm recovering, it's just really play by it by ear. Like I'm not dealing with fatigue and pain as the only symptoms of this thing. I also have, like an active wound drainage. So, it's like it's something I need to constantly manage. Male, UC, #003*

*But then it’s also not guaranteed if somebody is really struggling with constant flares then I think that would be really hard to maintain [a routine]. But yeah, if you're just in remission and been able to stay in remission, I think you can definitely maintain a program. Female, UC, #017*

*Coping strategies*: Most participants reported using coping strategies to improve engagement with PA. The number of coping strategies available to individuals tended to be related to the number of years since IBD diagnosis, that is, the longer individuals had lived with IBD, the more coping strategies they had developed. Coping strategies found to influence *physical capability* included getting optimum nutrition, good sleep, and performing PA during periods of high energy. All these impacted their motivation for PA; which highlights how improvement in 1 component of the COM impacts other components:

*Also like how well I’m eating is a is a big factor. . .if I’m eating well, I feel better. And so then I feel more motivated. Male, CD, #004*

*. . .like I have to. . .so like on Monday night, I have to sleep early so that I can wake up early Tuesday because I know I have to go for my activity class and I’ll be more energetic. So that’s the weakest point for me, the energy level one so I’ll get more energy by getting more sleep and I’ll be more active in my class. Female, UC, #009. Yeah. Like, last summer I was still working night shift at the time so when I had bursts of energy at 2 a.m., I’d go to my garage, I’d lock myself in, so I felt safe, and I’d workout in the garage. Female, CD, #015.*

Coping strategies that were found to influence *psychological capability* included having a positive attitude towards PA and being mindful of the limitations of living with IBD:

*Yeah, but I mean. . .there's a lot of mental to the physical part. And something I've been kind of coming to terms with is I'm not as capable as a normal person, and that there's nothing wrong with not reaching 20 (reps) if you achieved 25 (reps) last week. Right? Like there's ups and downs, that's kind of how the way the world goes. Male, UC, #003*

*Determination*: One important theme related to psychological capability was participants’ determination to perform PA despite challenges and barriers. This determination enhanced their ‘capability’ for PA engagement:

*I really don’t try and let my symptoms get in the way of my physical activity. Are some days harder than the other? Yes. Or some days I’m at the gym and some days I have a really bad fatigue and I’m just on the couch. But overall, I think that I do try and lead a very active lifestyle.*

*Because also I think going through the thick of my disease – I think coming out on the other side, I’m a lot more grateful I think – for what my body can do. So, I think that I really try and practice physical activity and I’m just more thankful for it. Female, CD, #012 Yeah. Every now and then like I’ll still get like a lot of bloating and there will be like a sharp pain from time to time in my stomach. But it’s really not enough to stop me from being active.And I don’t know, I’m a little bit like strong-willed in that way. Female, UC, #021*

**Opportunity:**

*We identified 4 themes that influenced ‘opportunity’, which refers to a favourable set of circumstances, for study participants. They were *social support, type of employment, bathroom access, and finances*.*

*Social support*: In their discussion, participants reported that living with IBD as adults requires that they balance living with a chronic condition with work, family, and school responsibilities. This sometimes limited opportunities for PA participation. However, participants reported that family members and friends could be sources of social support for PA engagement, thereby enhancing the opportunity for PA participation. In their discussion, participants highlighted that having this social support also improves their motivation to participate in PA:

*On the days when I didn't want to do it, my mom would push me and vice versa. But yeah, I feel like exercise programs are like, in theory they're great, but if you don't have anybody holding you accountable, there's nothing stopping you from just being like, ‘well, I don't feel like it today’, and the next day you're like, ‘Well, I still don't feel like it’. And then suddenly, it's been 3 weeks, and you haven't done it. Female, CD, #007.*

*My partner, she just got an Apple watch just after Christmas, and it like gamifies physical activity for you. So, I kind of piggybacked on her program. So, whenever she goes and works out I would workout, too, and that whole thing has been really motivating. Male, CD, #004 Definitely encouraging me is when other people are doing it, you can kind of do the same thing as them. Just like that P90X work out, you just kind of move along with the routine. Male, UC,#020*

*Type of employment*: Participants who had jobs that required a lot of physical activity throughout the day reported having opportunities for PA through their employment. They tended not to be willing to do PA outside of work because it required additional energy in the context of fatigue as a persistent symptom:

*I come home from work, and I’m so tired after work ‘cause I do physical work, I just want to sit down. I don’t want to, you know, go do a workout. Male, CD, #001*

*Bathroom Access*: The ease of bathroom access in workout spaces was found to affect opportunity for PA participation. All study participants highlighted the value of having access to bathrooms in work-out spaces, such as parks with bathrooms and gyms with private bathroom stalls, as this gave them peace of mind to participate. Lack of bathrooms limited ‘opportunity’ for PA:

*. . .mentally I have been in a state where I don’t want to exert much physical activity on myself. Because maybe I’m cautious that it will trigger something and if a bathroom is not available to me, I may need to run somewhere. And that has been at the back of my mind. I have realized lately that there’s always – that thought is always running in my mind. Male, UC, #014*

*Finances*: Most participants reported that the ability to afford equipment, gym memberships, or even set up a work-out space within their home enhanced opportunities for PA engagement:

*Yeah. I would say that being able to afford equipment has definitely helped with my activity. As I said, I have an exercise bike, I have free weights, I have bands, I have a squat rack in my garage Female, CD, #015.*

*For me, I’d probably just do like the [workout] videos, I suppose, because, yeah, budget is an issue. Male, UC, #013*

**Motivation:**

The themes that influenced ‘motivation’, that is, the complex psychological process that focuses on achieving pre-set goal(s), including both the initiation and maintenance of efforts to achieve the goal(s), were **awareness of the benefits of PA, knowledge of effects of PA on IBD, social supports, and personal goals with a system of tracking progress.**

*Awareness of the benefits of PA*: From the discussion, some participants believed that performing PA improved their quality of life. Performing PA made them feel happy, improved their self-confidence, and reduced the impact of fatigue. The desire to continue to gain these benefits enhanced their motivation to engage more in PA:

*Like I found that when I’m a little bit more fit, I’m a little bit happier. When I'm a little bit more fit, things just go a little bit easier. My movements are. . .my bowel movements are a little bit more regular. . . .and I think that that's desirable if you're kind of living with this condition. Male, CD, #004*

*I think it’s just. . .you have to do something that makes yourself feel good because I think – when I exercised more or was more physically active, I felt better. So it was just, “Oh. If I get active then I will feel that confidence again.” Female, UC, #011*

When discussing their experiences with PA engagement, participants highlighted that the day-to-day experience of living with persistent symptoms of IBD not only impacted the capability to engage in PA, but also reduced their motivation to participate. They highlighted that factors such as bloating, abdominal pain, frequent fatigue, and anxiety, often reduced their motivation for PA participation:

*Yeah, and I would say lack of motivation. But that's not necessarily due to the IBD. But I mean that also, I guess kind of ties in with the fatigue and, you know, the exhaustion that comes from Crohn’s. Female, CD, #007*

However, despite this, all participants expressed they were aware of the benefits of PA, and ideally wanted to perform more PA. Both males and females reported wanting gains related to better body image and weight loss:

*I saw a picture of myself that my mom took of me. And I was like I hate the way I look and I gotta go do CrossFit. And I just jumped in. It [PA] just kinda made the process faster in terms of getting rid of that whole moon face thing. I knew it was going to go away once I stop taking the drug but it was just really fast [with PA], and it felt great. Male, CD, #001.*

*And when you’re on Prednisone, technically your body goes into a fast remission too. So, you’re not really experiencing a lot of the – maybe the same symptoms. Obviously joint pain was huge and the weight gain was huge, but I think at that point – especially with that rapid weight gain – I wish there had been a physical program I could have relied on. Female, CD, #012*

*Knowledge*: Several participants highlighted a lack of information about PA and IBD, especially early on in their IBD journey. Participants also noted that information available to them about their disease and living with it were often in formats they could not easily understand. This increased their frustration with living with IBD and their anxiety with performing PA. The lack of information not only impacted their motivation to participate but also reduced their capability for participation:

*I think what's frustrating about getting those materials given to you at first is, I remember being given a book on it but then every chapter or every question you have, the answer’s like, “Well, we don’t know what causes this.” So there’s also no clear answer with IBD for any of the questions you have, so I think I feel like I gave up kind of quickly on searching for those answers. Female, UC, #017.*

Some participants further highlighted that if they could receive information about PA from trustworthy sources like their doctor or from an IBD organization/institution, it would lessen the anxiety around PA participation:

*So having that coordinated information because. . .and I think that’s what prevents a lot of people when they don't know what to do or how to do it; having somebody who is like, ‘hey, If you're like just starting out, or you've like lost a bunch of weight or muscle mass due to your condition, and everything hurts, what’s some like really low impact stuff that you can do that you can feel good about and not hurting your body?’ I feel like finding that for myself, that definitely would have helped me out. Male, CD, #004*

*. . .yeah, if I think I did hear my gastroenterologist say you have to be more physically active and here’s some things you can do, I think that would be a push for sure, a push to do it. Female, UC, #017.*

*Social Support*: When discussing their experiences, participants often spoke about how living with IBD tends to be isolating. They noted that IBD is rarely talked about and even when it is talked about, family, and friends do not often understand the challenges of living with IBD. The loneliness of living with IBD was found to lessen the motivation to engage in PA. However, as noted earlier, participants highlighted that the availability of a family member or friend who can participate in PA with them, or a support group involving other individuals living with IBD participating in PA, might motivate them to participate. Availability of social support not only provides ‘motivation’ to engage in PA, it also provides ‘opportunity’ for PA engagement:

*And I also found after my latest surgery in 2020, I was messaging with some girls – who are around my age – and I was asking them, “Oh, what do you do when you go to the gym?” And then they would tell me what they would do. And that’s how I got most of the answers, is from a support group. Female, UC, #011*

*And then also, I think if you build community, I think community holds you accountable. So, if you have a group of people you’re working out with consistently – and for four sessions you’re not showing up – I think those people are even going to start looking out for you too. Female, CD, #012*

*Personal goals*: Some participants found that setting personal PA goals, as well as the availability of a system of tracking progress towards achieving those goals, improved motivation for PA:

*But just tracking things like that sometimes can give you the happy feeling of being like,‘Oh, I achieved this, and it feels good. So, I’m going to keep trying to go like for like a streak’ or something like that. And you know, do so many days in a row. Female, CD, #007*

*I mean, I enjoy going to the gym, but tracking helps me with reaching my goals faster I would say. So, yeah like if I want to hit certain targets within certain time, then it’s definitely helpful. Male, UC, #010.*

**Sex differences in experiences with PA**

The thematic analysis revealed only a few sex-related differences in the experiences of PA among study participants. Specifically, **social context of workout spaces and safety concerns.**

*Social context of workout spaces*: The report of being self-conscious about how they are seen or perceived when in workout spaces, especially gyms, was identified only among some of the female participants:

*But when I’m at the gym, I’m not only focused on the exercise, I am also focused on if my top is lifting, if my bag is showing. . .so then that makes me not want to spend money on a gym membership to go somewhere where you’re going to get looked at. Female, UC, #011.*

The perception individuals have about their workout spaces may influence their motivation to perform PA in those spaces.

*Safety*: Females more than males expressed safety concerns when considering options of workout locations. Thus, there might be fewer options available to females when they want to engage in PA compared to males, which may consequently influence levels of engagement between both sexes:

*I like to do my walks in the evenings. But I also find too there's the aspect of safety. Right? And that's the thing. I'm not gonna go if it's getting dark, you know. I feel like I'd be fine, but you never know. And that's something as women, we also have to think about it. Female, CD, #007.*

These themes, social context of workout spaces and safety concerns, may impact the ‘opportunity’ and ‘motivation’ for PA engagement for females but not for males. Thus, women may require different PA engagement options and strategies from men.

## Discussion

In this paper, we explored the experiences of PA engagement among adults with quiescent IBD to understand the low PA participation, as noted in previous research among members of this population. From the experiences of participants, we elicited the factors influencing PA engagement while living with quiescent IBD. We used the COM-B model, which proposes that a desired behaviour (B) is possible when capability (C), opportunity (O), and motivation (M) are present; to organize the study data. From the participants’ experiences, we found that presence of persistent symptoms, PA engagement prior to IBD diagnosis, maintaining a PA routine while living with IBD, coping strategies and determination influenced ‘capability’ for PA participation. Social support, type of employment, bathroom access, and finances influenced ‘opportunity’. Having an awareness of the benefits of PA while living with IBD, knowledge of the effects of PA on IBD, social support, and personal goals promoted ‘motivation’ for PA participation. We also explored sex differences in the experiences and perspectives of study participants. We found that all participants expressed the desire to be more physically active. However, females expressed concerns pertaining to the social context of work out spaces and safety issues.

From participants’ experiences with PA engagement, we elicited barriers to and facilitators of PA participation. Barriers included ongoing IBD-related persistent symptoms, low or no PA engagement prior to IBD diagnosis, lack of social support, poor bathroom access, lack of financial support, and lack of knowledge on PA engagement while living with IBD. Factors that can facilitate PA engagement include being physically active prior to IBD diagnosis, development of coping strategies, presence of social support, optimal bathroom access in workout spaces, employment that involves lots of physical activity, availability of relevant knowledge on PA, adhering to a PA routine, and ability to track progress achieved via gadgets and apps.

PA has been reported to be potentially beneficial for individuals with quiescent IBD.^3,27,44^ It is been hypothesized that during exercise, interleukin 6 (IL-6) is released from muscles and this exerts an anti-inflammatory effect in those with chronic inflammation, as IL-6 inhibits TNF production and enhance the stimulation of IL-1ra and IL-10.^45^ The full extent of the benefits of PA is yet to be elucidated as research on this subject is quite sparse. In-depth research may be challenging with the lack of evidence-informed PA and exercise guidelines for individuals with IBD. When healthcare providers and individuals with IBD have the necessary guidelines for PA promotion and engagement, research on this subject will hopefully become more homogenous, and objective PA data to assess the effects of PA and exercise on IBD can be derived. Our study aimed to bridge this gap by identifying barriers to PA engagement, and highlighting relevant factors to consider to promote PA engagement among individuals with IBD.

The promotion of PA engagement can begin as soon as a diagnosis of IBD is made. Gastroenterologists are in an excellent position to encourage patients to engage in PA, dispel concerns they may have about PA engagement, and make referrals to resources to help patients find their own optimum levels of PA engagement. In a 2019 UK study on PA engagement following stoma surgery, 49% reported not receiving any information or advice about PA.^46^ Similarly, in an Italian cross-sectional study on barriers to PA participation by individuals with quiescent IBD, less than 50% of respondents reported receiving appropriate support from family or gastroenterologists about PA participation. Knowledge on the different types of PA that are beneficial when living with IBD is crucial to promote PA participation.^28^ Participants in our study emphasized the value of getting knowledge on PA and recommendations for participation from a trusted professional, especially their gastroenterologists as a factor that would enhance both their motivation and capability to participate more in PA. In agreement, participants in the qualitative study of Wang and colleagues^25^ emphasized the need for professional guidance for PA engagement. Similarly, a 2022 narrative review concluded that it might be beneficial for physicians to discuss PA programs with their patients especially those dealing with fatigue, depression, anxiety, or impaired QoL.^46^

Interestingly, we found that some participants demonstrated determination to participate consistently in PA despite barriers to engagement. Our conceptualization of this theme is that participants demonstrated determination to regain a sense of control over the unpredictability of living with IBD by maintaining a PA routine, that is, something they could control. Study themes reveal a thread of acceptance and flexibility in living with IBD. This state of acceptance could be leveraged to promote PA engagement via targeted interventions. As highlighted in previous studies,^47,48^ adults with IBD strive for normalcy in their everyday lives. Being able maintain control over IBD by regularly engaging in PA can help people achieve this normalcy. Targeted interventions to promote PA engagement identified in our study included promotion of PA participation by gastroenterologists and other health care providers by providing knowledge. As well as leveraging family and friends for social supports, developing individualized coping strategies (such as good nutrition, optimum sleep, maximizing periods of high energy), setting personal goals and using gadgets and apps to monitor PA engagement.

Study findings revealed that when participants expended effort, time, and energy to engage in PA, they found it gratifying to observe the gains, not just in terms of improvement in quality of life, but also the quantification of their progress and achievements. This enhanced their motivation to maintain their PA participation. In the study of Matilla et al,^49^ they used mobile applications in an intervention to promote healthy lifestyle changes in adults with elevated health risks. They reported that participants valued having a record of their personal progress in real time, observable health outcomes (such as weight loss), and being provided with a personalized exercise plan. Incorporating a system that provides regular feedback may encourage performance and maintenance of PA. With the proliferation of digital health in IBD management,^50-52^ this might be just the right time for healthcare providers to encourage the use of wearable devices in PA engagement among this group.

A 2024 cross-sectional study investigating the behaviours and barriers to exercise in IBD found engagement was significantly lower among female participants compared to males.^28^ We found in our study that the social context of workout spaces and safety concerns may impact PA engagement for females but not so much for males. As such, females with quiescent IBD may experience more barriers to PA engagement than their male counterparts. This suggests that females will require additional supports to participate in PA and may require different PA engagement options and strategies (e.g. prioritizing social support, working out in groups) from males.

## Strengths and Limitations

Previous research has highlighted the low PA participation among adults with quiescent IBD.^13,19,30^ But research to improve our understanding of this PA behaviour, as well as facilitators to promote behaviour change is lacking. This study provides highly relevant data to fill this gap. Additionally, this study provides patient perspectives in this area of research, to ensure future research incorporates patients’ preferences. Furthermore, using COM-B model to organize our study findings not only enhances the understanding of PA behaviour among those with quiescent IBD; it also provides a comprehensive theoretical framework to underpin the development of effective, theory-based interventions. A limitation of this study is that only adults between the ages of 20 and 45 years old were included. The perspectives of younger and older participants might provide a more comprehensive understanding of aspects that influence PA behaviour in different age groups. Although we used a systematic approach to determine when to stop recruitment, it is possible that the full range of perspectives and experiences were not included. Our use of videoconferencing software may have limited participation from people who did not have access to the necessary technology. We acknowledge that COM-B coding is a subjective process and there may be different ways of coding the findings. In our analysis, we tailored the COM-B coding to our study objectives, which may not be applicable to other research studies. Despite these limitations, this study provides relevant information for promoting PA behaviour change in the context of living with IBD.

## Conclusions

This study provides more clarity on the factors that influence PA behaviour among adults with quiescent IBD. We have highlighted how the COM-B model can help organize these factors to identify the aspects that influence each component of the COM to promote PA behaviour change. Consistent PA participation may serve as an adjunctive management for the psychological aspects of IBD, which historically receive less attention than physical symptoms.^53^ We recommend that clinicians should actively promote PA engagement among their patients with IBD because the patients may need the knowledge on PA and reassurance that participating in PA is safe for them.

## Supplemental Material

sj-docx-1-rpo-10.1177_27536351251382074 – Supplemental material for Behaviour Change Considerations to Promote Physical Activity Participation among Individuals with Quiescent Inflammatory Bowel Disease: Barriers and Facilitators Supplemental material, sj-docx-1-rpo-10.1177_27536351251382074 for Behaviour Change Considerations to Promote Physical Activity Participation among Individuals with Quiescent Inflammatory Bowel Disease: Barriers and Facilitators by Banke Oketola, Sandra Webber, Harminder Singh, Maia Kredentser, Kristin Reynolds and Gayle Restall in Advances in Rehabilitation Science and Practice
