# Supplementary material for: Behaviour Change Considerations to Promote Physical Activity Participation among Individuals with Quiescent Inflammatory Bowel Disease: Barriers and Facilitators
Source: Adv Rehabil Sci Pract. 2025 Oct 20;14:27536351251382074. doi: 10.1177/27536351251382074 (PMC12550254; doi:10.1177/27536351251382074)
Supplement: sj-docx-1-rpo-10.1177_27536351251382074 – Supplemental material for Behaviour Change Considerations to Promote Physical Activity Participation among Individuals with Quiescent Inflammatory Bowel Disease: Barriers and Facilitators [file sj-docx-1-rpo-10.1177_27536351251382074.docx]

APPENDIX I

Consolidated criteria for reporting qualitative studies (COREQ): 32-item checklist

For: Behaviour Change Considerations to Promote Physical Activity Participation among Individuals with Quiescent Inflammatory Bowel Disease – Barriers and Facilitators

Tong A, Sainsbury P, Craig J. Consolidated criteria for reporting qualitative research (COREQ): a 32-item checklist for interviews and focus groups. International Journal for Quality in Health Care. 2007. Volume 19, Number 6: pp. 349 – 357

| **Item No** | | **Guide Questions/Description** | **Reported on Page #** |  |
| --- | --- | --- | --- | --- |
| **Domain 1: Research team and reflexivity** | | | |  |
| **Personal Characteristics** | | | |  |
| 1. Interviewer/ facilitator | | Banke Oketola | Pg. 7 |  |
| 2. Credentials | | MSc | Pg. 1 |  |
| 3. Occupation | | Clinical Research Coordinator | Not reported. |  |
| 4. Gender | | Female | Not reported. |  |
| 5. Experience and training | | Qualitative research courses at MSc. and PhD levels, 2 previous qualitative research projects; performed data collection, analysis and reporting. | Not reported. |  |
| **Relationship with participants** | | | |  |
| 6. Relationship established | | No | N/A |  |
| 7. Participant knowledge of the interviewer | | Interviewer’s credentials were shared with participants in the informed consent form. Goals for doing the research were shared at the interview. | N/A |  |
| 8. Interviewer characteristics | | Participants were informed that the interviewer is a Physical therapist by training and worked in IBD research for over 6 years. Interest and bias, which is to bring physical therapy into IBD research were also shared with some participants during the interviews. | N/A |  |
| **Domain 2: study design** | | |  |  |
| **Theoretical framework** | | |  |  |
| 9.Methodological orientation and Theory | Qualitative description | Pg. 5 |  |  |
| **Participant selection** | | |  |  |
| 10. Sampling | Purposive sampling | Pg. 6 |  |  |
| 11. Method of approach | Telephone | Pg. 7 |  |  |
| 12. Sample size | 15 | Pg. 10 |  |  |
| 13. Non-participation Setting | N/A  Reasons include scheduling conflicts and not meeting eligibility criteria. | N/A |  |  |
| 14. Setting of data collection | Zoom | Pg. 7 |  |  |
| 15. Presence of nonparticipants | No | N/A |  |  |
| 16. Description of sample | Provided. | Pg. 9 |  |  |
| **Data collection** | | |  | No |
| 17. Interview guide | Semi-structured interview guide was used.  It was pilot tested. | Pg. 7 |  |  |
| 18. Repeat interviews | None | N/A |  |  |
| 19. Audio/visual recording | Video-recording | Pg. 7 |  |  |
| 20. Field notes | None | N/A |  |  |
| 21. Duration | 35-60 minutes. | Pg. 10 |  |  |
| 22. Data saturation | Approach to data saturation provided. | Pg. 7 |  |  |
| 23. Transcripts returned | Summary of findings were provided to participants for review and feedback. | Pg. 9 |  |  |
| **Domain 3: analysis and findings** | | |  |  |
| **Data analysis** | | |  |  |
| 24. Number of data coders | 2 | Pg. 8 |  |  |
| 25. Description of the coding tree | Coding and levels of themes described. | Pg. 8 |  |  |
| 26. Derivation of themes | Derived in advance | Pg. 8 |  |  |
| 27. Software | NVivo Qualitative data analysis software by Lumivero | Pg. 8 |  |  |
| 28. Participant checking | Participants provided feedback on study summary. | Pg. 9 |  |  |
| **Reporting** | | |  |  |
| 29. Quotations presented | Participants’ quotations were presented to buttress themes and findings. Each quotation was identified by gender, IBD diagnosis and participant ID. | Pg 10 - 19 |  |  |
| 30. Data and findings consistent | Consistent | Pg 10 - 24 |  |  |
| 31. Clarity of major themes | Major themes were clearly presented in the results section using headings. | Pg 10 - 19 |  |  |
| 32. Clarity of minor themes | A description of minor themes and unexpected themes were provided using subheadings. | Pg 10 - 19 |  |  |

APPENDIX II

Quotes Table

Experiences with PA participation:

| Domain | Themes | Statements |
| --- | --- | --- |
| Capability | Persistent symptoms | *“Whether you are doing push ups or running, or even walking, you always use your core muscles. And I guess if you have Crohn’s or colitis, you already have more pain there.” Male, CD, #001*  *“I guess my fatigue is definitely something that I find is very challenging to overcome. Fatigue is a big one… It’s something that you always have to fight through.” Female, CD, #012*  *“And it's a lot, I mean I feel like it's just the same with like living with IBD in general, like it's a lot of trial and error in things like, whether it's food, or whether it's exercise or anything like that. And like sometimes trial and error gets to be too much. So, sometimes you just have to…take a step back.” Famale, CD, #007.* |
|  | PA engagement prior to IBD diagnosis | *“Well, before I was like sick, I was pretty physical. So, I managed to have like a good starting point for when I was recovering…I didn't find it challenging to get back into exercising. I think. Yeah, I mean it wasn't too hard for me.” Man, CD, #001*  *“No, I think at the start of everything you need a certain person, like a professional’s advice. I don’t want to do online exercises and the next day I’ll be like, oh, my back hurts. So, I just want to get a professional advice at the start and then I’ll make my to-do list.” Female, UC, #009* |
|  | Maintaining a PA routine | *“Yeah, I think to get back into it, even just walking, there wasn't a lot of anxiety.” Female, CD, #005.*  *“I think the problem currently with my routine is like the way I'm recovering, it's just really play by it by ear. Like I'm not dealing with fatigue and pain as the only symptoms of this thing. I also have, like an active wound drainage. So, it's like it's something I need to constantly manage.” Male, UC, #003*  *“But then it’s also not guaranteed if somebody is really struggling with constant flares then I think that would be really hard to maintain [a routine]. But yeah, if you're just in remission and been able to stay in remission, I think you can definitely maintain a program.” Female, UC, #017* |
|  | Coping strategies | *“Also like how well I’m eating is a is a big factor…if I’m eating well, I feel better. And so then I feel more motivated.” Male, CD, #004*  *“…like I have to…so like on Monday night, I have to sleep early so that I can wake up early Tuesday because I know I have to go for my activity class and I’ll be more energetic. So that’s the weakest point for me, the energy level one so I’ll get more energy by getting more sleep and I’ll be more active in my class.” Female, UC, #009.*    *“Yeah. Like, last summer I was still working night shift at the time so when I had bursts of energy at 2 a.m., I’d go to my garage, I’d lock myself in, so I felt safe, and I’d workout in the garage.” Female, CD, #015.* |
|  | Determination | *“I really don’t try and let my symptoms get in the way of my physical activity. Are some days harder than the other? Yes. Or some days I’m at the gym and some days I have a really bad fatigue and I’m just on the couch. But overall, I think that I do try and lead a very active lifestyle. Because also I think going through the thick of my disease – I think coming out on the other side, I’m a lot more grateful I think – for what my body can do. So, I think that I really try and practice physical activity and I’m just more thankful for it.” Female, CD, #012*  *“Yeah. Every now and then like I’ll still get like a lot of bloating and there will be like a sharp pain from time to time in my stomach. But it’s really not enough to stop me from* *being active. And I don’t know, I’m a little bit like strong-willed in that way.” Female, UC, #021* |
| Opportunity | Social Support | *“On the days when I didn't want to do it, my mom would push me and vice versa. But yeah, I feel like exercise programs are like, in theory they're great, but if you don't have anybody holding you accountable, there's nothing stopping you from just being like, ‘well, I don't feel like it today’, and the next day you're like, ‘Well, I still don't feel like it’. And then suddenly, it's been 3 weeks, and you haven't done it.” Female, CD, #007*  *“My partner, she just got an Apple watch just after Christmas, and it like gamifies physical activity for you. So, I kind of piggybacked on her program. So, whenever she goes and works out I would workout, too, and that whole thing has been really motivating.” Male, CD, #004*  *“Definitely encouraging me is when other people are doing it, you can kind of do the same thing as them. Just like that P90X work out, you just kind of move along with the routine.” Male, UC, #020* |
|  | Type of employment | *“I come home from work, and I’m so tired after work ‘cause I do physical work, I just want to sit down. I don’t want to, you know, go do a workout.” Male, CD, #001* |
|  | Bathroom access | *“…mentally I have been in a state where I don’t want to exert much physical activity on myself. Because maybe I’m cautious that it will trigger something and if a bathroom is not available to me, I may need to run somewhere. And that has been at the back of my mind. I have realized lately that there’s always – that thought is always running in my mind.” Male, UC, #014* |
|  | Finances | *“Yeah. I would say that being able to afford equipment has definitely helped with my activity. As I said, I have an exercise bike, I have free weights, I have bands, I have a squat rack in my garage” Female, CD, #015.*  *“For me, I'd probably just do like the [workout] videos, I suppose, because, yeah, budget is an issue.” Male, UC, #013* |
| Motivation | Awareness of the benefits of PA | *“Yeah, and I would say lack of motivation. But that's not necessarily due to the IBD. But I mean that also, I guess kind of ties in with the fatigue and, you know, the exhaustion that comes from Crohn’s.” Female, CD, #007*  *“I saw a picture of myself that my mom took of me. And I was like I hate the way I look and I gotta go do CrossFit. And I just jumped in. It [PA] just kinda made the process faster in terms of getting rid of that whole moon face thing. I knew it was going to go away once I stop taking the drug but it was just really fast [with PA], and it felt great. Male, CD, #001.*  *“And when you’re on Prednisone, technically your body goes into a fast remission too. So, you’re not really experiencing a lot of the – maybe the same symptoms. Obviously joint pain was huge and the weight gain was huge, but I think at that point – especially with that rapid weight gain – I wish there had been a physical program I could have relied on.” Female, CD, #012*  *“Like I found that when I’m a little bit more fit, I’m a little bit happier. When I'm a little bit more fit, things just go a little bit easier. My movements are…my bowel movements are a little bit more regular. …and I think that that's desirable if you're kind of living with this condition.” Male, CD, #004*  *“I think it’s just…you have to do something that makes yourself feel good because I think – when I exercised more or was more physically active, I felt better. So it was just, “Oh. If I get active then I will feel that confidence again.” Female, UC, #011* |
|  | Knowledge of effects of PA on IBD | *“I think what's frustrating about getting those materials given to you at first is, I remember being given a book on it but then every chapter or every question you have, the answer’s like, “Well, we don’t know what causes this.” So there’s also no clear answer with IBD for any of the questions you have, so I think I feel like I gave up kind of quickly on searching for those answers.” Female, UC, #017.*  *“So having that coordinated information because…and I think that’s what prevents a lot of people when they don't know what to do or how to do it; having somebody who is like, ‘hey, If you're like just starting out, or you've like lost a bunch of weight or muscle mass*  *due to your condition, and everything hurts, what’s some like really low impact stuff that you can do that you can feel good about and not hurting your body?’ I feel like finding that for myself, that definitely would have helped me out.” Male, CD, #004*  *“…yeah, if I think I did hear my gastroenterologist say you have to be more physically active and here’s some things you can do, I think that would be a push for sure, a push to do it.” Female, UC, #017.* |
|  | Social support | *“And I also found after my latest surgery in 2020, I was messaging with some girls – who are around my same age – and I was asking them, “Oh, what do you do when you go to the gym?” And then they would tell me what they would do. And that’s how I got most of the answers, is from a support group.” Female, UC, #011*  *“And then also, I think if you build community, I think community holds you accountable. So, if you have a group of people you’re working out with consistently – and for four sessions you’re not showing up – I think those people are even going to start looking out for you too.” Female, CD, #012* |
|  | Personal goals with a system of tracking progress. | *“But just tracking things like that sometimes can give you the happy feeling of being like, ‘Oh, I achieved this, and it feels good. So I’m going to keep trying to go like for like a streak’ or something like that. And you know, do so many days in a row.” Female, CD, #007*  *“I mean, I enjoy going to the gym, but tracking helps me with reaching my goals faster I would say. So, yeah like if I want to hit certain targets within certain time, then it’s definitely helpful.” Male, UC, #010.* |

Sex Differences:

| Themes | Statements |
| --- | --- |
| Social context of workout spaces | *“But when I’m at the gym, I’m not only focused on the exercise, I am also focused on if my top is lifting, if my bag is showing…so then that makes me not want to spend money on a gym membership to go somewhere where you’re going to get looked at.” Female, UC, #011.* |
| Safety concerns | *“I like to do my walks in the evenings. But I also find too there's the aspect of safety. Right? And that's the thing. I'm not gonna go if it's getting dark, you know. I feel like I'd be fine, but you never know. And that's something as women, we also have to think about it.” Female, CD, #007.* |
